# Single dominant right coronary artery with hypoplastic left coronary branch: a rare congenital anomaly: a case report

**DOI:** 10.1093/ehjcr/ytae017

**Published:** 2024-01-08

**Authors:** Fadhel Hamidani, Blerim Berisha

**Affiliations:** Cardiology Department, Clinic Neuendettelsau, Heilsbronner Str. 44, Neuendettelsau 91564, Germany; Cardiology Department, Clinic Neuendettelsau, Heilsbronner Str. 44, Neuendettelsau 91564, Germany

**Keywords:** Single coronary artery, Hypoplastic coronary artery, Sudden cardiac death, Case report

## Abstract

**Background:**

A right single coronary artery (SCA) and hypoplastic coronary disease represent a rare coronary artery anomaly, which is associated with sudden cardiac death. The clinical manifestations of these anomalies depend on the distribution of collateral vessels.

**Case summary:**

A 55-year-old female presented with dyspnoea, mild chest pain during physical activity, and palpitations. Selective coronary angiography revealed a prominent SCA originating from the right coronary sinus. Approximately 2 mm from the ostium, this artery branched into two: a dominant right coronary artery (RCA) and a smaller artery for the anterior wall perfusion. The dominant RCA then moved posteriorly, bifurcating into a posterior descending artery and a posterolateral artery. The latter occupied the expected location of the left circumflex artery and supplied the majority of the coronary circulation, including the left ventricle. Notably, there were no significant atherosclerotic calcifications or stenoses observed.

**Discussion:**

We describe a unique case of a SCA that doesn’t conform to any category within the modified Lipton’s classification. Symptoms are speculated to arise from the compression of the SCA between the aorta and pulmonary artery during physical exertion. Additionally, hypoperfusion from the hypoplastic left coronary branch in the anterior wall warrants consideration. It’s paramount to meticulously evaluate the risk of sudden cardiac death when treating patients with a SCA. For high-risk patients, coronary artery bypass grafting should be contemplated.

For our patient, given the clinical context, pharmacological treatment optimization was chosen.

**Learning points**

- Angiographic description and multimodality imaging are required in the diagnosis and management of single coronary disease.
- In the treatment of patient with single coronary artery is essential to carefully evaluate the risk of sudden cardiac death.
- In patients with high risk, a coronary artery bypass grafting should be considered.

## Introduction

Single coronary artery (SCA) is a rare coronary artery anomaly in medical literature. It is usually diagnosed incidentally during coronary artery angiography or post-mortem following sudden cardiac death.^1,2^ The angiographic classification of SCA was proposed by Lipton *et al.*^3^ and was later modified by Yamanaka *et al.*^4^ Hypoplastic coronary artery disease is another rare congenital anomaly associated with sudden cardiac death, reported in 2.2% of patients with coronary anomalies.^5^ Aubry *et al.*^6^ proposed a simplified classification for proximal anomalous connections of the coronary arteries.

## Summary figure

**Figure:**
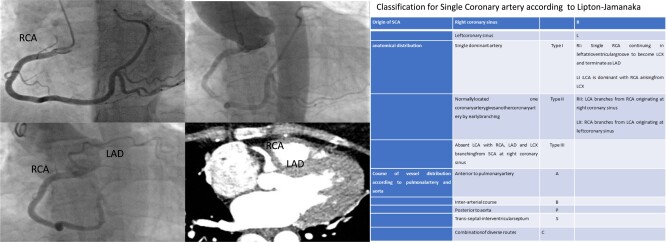
A 55-year-old female presented with dyspnoea and light chest pain during physical activity and palpitations. Coronary angiography and cardio-CT revealed a rare congenital anomaly: single dominant right coronary artery (RCA) with hypoplastic left coronary branch, which does not fit any of the types of the modified Lipton’s classification.

Clinical symptoms of coronary anomalies depend on collateral vessel distribution. We present a unique case of a SCA that does not conform to any type in the modified Lipton’s classification.

## Case presentation

**Figure 1:**
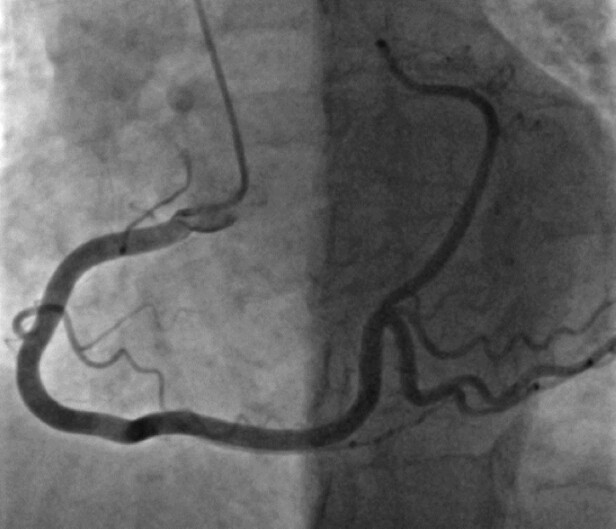
Dominant RCA bifurcates into PDA and posterolateral artery, traveling in the expected location of LCX.

**Figure 2:**
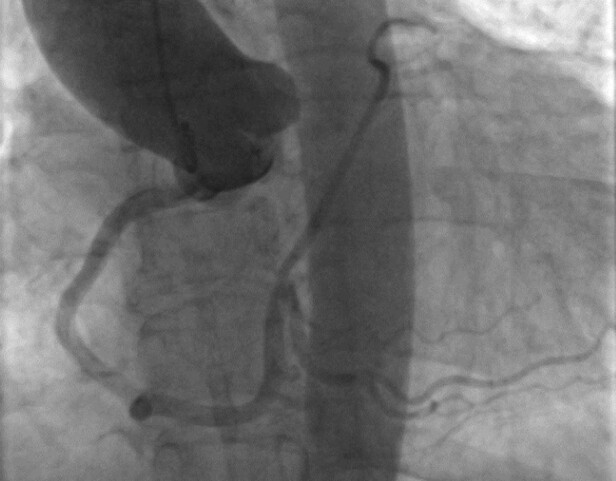
Large single coronary artery (SCA-R) shown in aortography with the absence of left main (LM).

A 55-year-old female presented with dyspnoea, mild chest pain during physical activity, and palpitations. We reviewed her medical history, clinical course, physical examination, laboratory findings, treatments, and current condition. Her past medical history indicated high blood pressure and elevated blood lipid levels. The electrocardiogram showed frequent supraventricular premature beats. Echocardiography revealed normal left ventricular function without wall motion abnormalities. We performed a coronary angiography, which showed a single, large coronary artery arising from the right coronary sinus (*Figures 1* and *2*).

**Figure 3:**
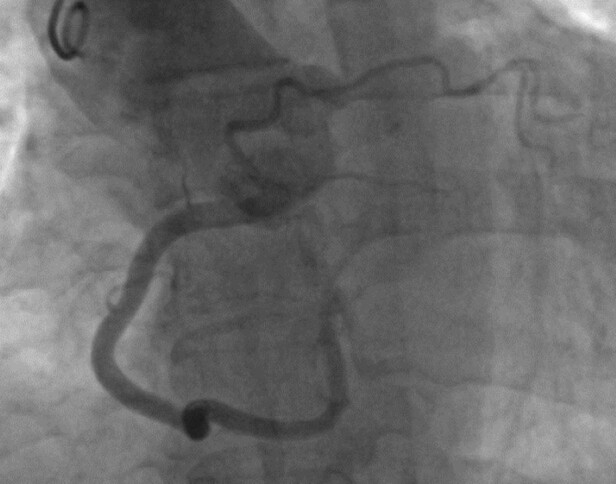
Hypoplastic left anterior descending artery (LAD) originating from the dominant right coronary artery (RCA).

**Figure 4:**
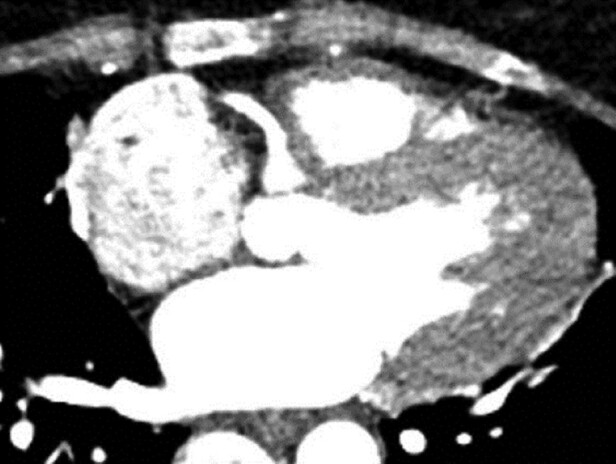
Hypoplastic left anterior descending artery (LAD) originating from the dominant right coronary artery (RCA).

The anterior wall perfusion is provided by one small branch originating from the initial part of the RCA in the absence of the left main (*Figure 3*). The dominant RCA branch travels posteriorly, bifurcating into a posterior descending artery (PDA) and a posterolateral artery branch, which is located where the left circumflex (LCX) is typically found. This branch supplies most of the coronary circulation, including the left ventricle (*Figure 1*). There were no significant atherosclerotic calcifications or stenosis. A computed tomography angiography examination was conducted using a Siemens-Somatom CT device from Germany. The SCA arises from the right coronary sinus. Approximately 2 mm from the ostium, this artery divides into two branches: a dominant RCA and a smaller branch responsible for the anterior wall perfusion (*Figure 4*). Stress echocardiography, conducted in an ambulatory setting, revealed no significant wall motion abnormalities, and no inducible arrhythmias were observed. A cardiac MRI was not possible due to the patient’s claustrophobia.

Based on the results of invasive and non-invasive diagnostics, we decided not to qualify the patient for invasive treatment. Due to our patient’s clinical situation, we decided to optimize pharmacological treatment. The following pharmacotherapy was recommended: atorvastatin 40 mg, amlodipine 2.5 mg, and acetylsalicylic acid 100 mg, and β-blocker therapy was discontinued because of temporary sinus bradycardia. Regular cardiological monitoring and risk factor reduction were recommended.

## Discussion

The incidence of a SCA is ∼0.04–1%.^1,2^ The classification of SCA was proposed by Lipton *et al.*^3^ and later modified by Yamanaka *et al.*^4^ This classification is based on the origin of the ostium from the Valsalva sinus: R for right and L for left. Roman numerals I, II, and III describe the anatomical distribution of the vessel, while letters A, B, P, S, and C detail the vessel’s course in relation to the pulmonary artery and aorta (*Table 1*). Type RI, according to this classification, is one of the rarest forms of SCA. In our case, the right SCA travelled posteriorly, bifurcating into the PDA and the posterolateral artery branch, positioned in the expected location of the LCX. However, the perfusion of the anterior wall was supplied by a small hypoplastic branch originating from the early part of the RCA, with the left main being absent. Our case does not align with any type from the modified Lipton’s classification. Aubry *et al.* introduced a simplified classification featuring eight types of proximal anomalous connections of the coronary arteries. In our scenario, the small hypoplastic anomalous artery connects with the contralateral artery (Type II).

**Table 1:** Classification for single coronary artery according to Lipton–Yamanaka

Origin of SCA	Right coronary sinus		R
	Left coronary sinus		L
Anatomical distribution	Single dominant artery	Type I	RI: single RCA continuing in left atrioventricular groove to become LCX and terminate as LAD LI: LCA is dominant with RCA arising from LCX
	Normally located one coronary artery gives another coronary artery by early branching	Type II	RII: LCA branches from RCA originating at right coronary sinus LII: RCA branches from LCA originating at left coronary sinus
	Absent LCA with RCA, LAD and LCX branching from SCA at right coronary sinus	Type III	
Course of vessel distribution according to pulmonal artery and aorta	Anterior to pulmonary artery	A	
	Inter-arterial course	B	
	Posterior to aorta	P	
	Trans-septal-interventricular septum	S	
	Combination of diverse routes	C	

Clinical outcomes for this group of patients vary significantly. While many patients with coronary anomalies are asymptomatic, others may experience chest pain, syncope, ventricular tachycardia, or sudden cardiac death during exercise. According to the Sudden Death Committee of the American Heart Association, coronary anomalies account for 19% of deaths in this patient group.^5,7^

It has been reported that patients with a SCA originating from the right coronary sinus are at a higher risk than those where the SCA originates from the left coronary sinus.^8^

Our patient presented with dyspnoea and mild chest pain during physical activity, but no significant coronary stenosis was found.

The pathophysiological mechanisms behind dyspnoea and chest pain in patients without coronary stenosis are not well understood. Microvascular circulation depends on a SCA, so any functional obstruction of this artery may be poorly tolerated. This obstruction is believed to occur due to the compression of the SCA between the aorta and the pulmonary artery during exercise.^9,10^ In our case, hypoperfusion from a small hypoplastic left coronary branch in the anterior wall should be considered. However, stress echocardiography did not reveal any significant wall motion abnormalities or inducible arrhythmias.

In the treatment of patients with a SCA, it is essential to evaluate the risk of sudden cardiac death carefully. For those at high risk, coronary artery bypass grafting (CABG) should be considered. In our case, a CABG left internal mammary artery to LAD is not feasible due to the very small diameter of the hypoplastic LAD. However, if there’s progressive atherosclerosis, a CABG to the main RCA should be considered.

## Data Availability

The data that support the findings of this case report are available from the corresponding author, upon reasonable request.

## Lead author biography

Dr Fadhel Hamidani is working as a cardiologist in Cardiology Department, Clinic Neuendettelsau, Germany. His speciality is invasive cardiology, cardiac devices, and cardiac imagine.

**Consent:** Written informed consent was obtained from the patient for publication of data and accompanying images according to COPE recommendations.

**Funding:** None declared.
